# Multifunctional redox modulator prevents blast-induced loss of cochlear and vestibular hair cells and auditory spiral ganglion neurons

**DOI:** 10.1038/s41598-024-66406-1

**Published:** 2024-07-03

**Authors:** Dalian Ding, Senthilvelan Manohar, Peter F. Kador, Richard Salvi

**Affiliations:** 1 Center for Hearing and Deafness, University at Buffalo, Buffalo, NY 14214 USA; 2 Therapeutic Vision, Inc., Omaha, NE 68164 USA

**Keywords:** Drug discovery, Neuroscience, Neurology

## Abstract

Blast wave exposure, a leading cause of hearing loss and balance dysfunction among military personnel, arises primarily from direct mechanical damage to the mechanosensory hair cells and supporting structures or indirectly through excessive oxidative stress. We previously reported that HK-2, an orally active, multifunctional redox modulator (MFRM), was highly effective in reducing both hearing loss and hair cells loss in rats exposed to a moderate intensity workday noise that likely damages the cochlea primarily from oxidative stress versus direct mechanical trauma. To determine if HK-2 could also protect cochlear and vestibular cells from damage caused primarily from direct blast-induced mechanical trauma versus oxidative stress, we exposed rats to six blasts of 186 dB peak SPL. The rats were divided into four groups: (B) blast alone, (BEP) blast plus earplugs, (BHK-2) blast plus HK-2 and (BEPHK-2) blast plus earplugs plus HK-2. HK-2 was orally administered at 50 mg/kg/d from 7-days before to 30-day after the blast exposure. Cochlear and vestibular tissues were harvested 60-d post-exposure and evaluated for loss of outer hair cells (OHC), inner hair cells (IHC), auditory nerve fibers (ANF), spiral ganglion neurons (SGN) and vestibular hair cells in the saccule, utricle and semicircular canals. In the untreated blast-exposed group (B), massive losses occurred to OHC, IHC, ANF, SGN and only the vestibular hair cells in the striola region of the saccule. In contrast, rats treated with HK-2 (BHK-2) sustained significantly less OHC (67%) and IHC (57%) loss compared to the B group. OHC and IHC losses were smallest in the BEPHK-2 group, but not significantly different from the BEP group indicating lack of protective synergy between EP and HK-2. There was no loss of ANF, SGN or saccular hair cells in the BHK-2, BEP and BEPHK-2 groups. Thus, HK-2 not only significantly reduced OHC and IHC damage, but completely prevented loss of ANF, SGN and saccule hair cells. The powerful protective effects of this oral MFRM make HK-2 an extremely promising candidate for human clinical trials.

## Introduction

In industrialized societies, noise is ubiquitous and a major cause of hearing loss among the young and middle aged^1,2^. Noise-induced hearing loss (NIHL), together with tinnitus and hyperacusis comorbidities, are extremely common among military personnel exposed to continuous, impulse or blast wave noise^3–5^. Hearing protection (e.g., earplugs/ear muffs), when worn properly, reduces the risk of NIHL^6^. However, hearing protection is seldom worn in front line combat or places where situational awareness and/or auditory communication are critical (e.g., emergency responders, call center operators)^7^. In many situations, explosions and gun fire occur unexpectedly before hearing protection can be deployed^7,8^.

Advances in understanding the biological bases of NIHL have led to pharmacologic efforts to reduce the risk of hearing losses induced mainly by the generation of free radicals and oxidative stress^9–14^. Free radical levels reportedly increase in marginal cells of the stria vascularis minutes to hours following intense noise exposure^15^. Because this early rise in oxidative stress occurs prior to significant hair cell loss, hearing loss was thought to result from other factors besides oxidative stress. However, subsequent research revealed a gradual and prolonged buildup of free radical induced oxidative stress in the organ of Corti and hair cells with levels peaking 7–10 days post-exposure followed by a decline, but with levels still noticeable 14 days post-exposure^16^. While the increase in free radicals is associated with hair cell degeneration, it is unclear if excess oxidative stress plays a major role in spiral ganglion neuron (SGN) degeneration.

Recently, we investigated whether HK-2 (1-(5-hydroxypyrimidin-2-yl) pyrrolidine-2, 5-dione), a novel multifunctional redox modulator (MFRM), could protect against NIHL. HK-2, which crosses the blood–brain barrier, not only scavenges free radicals, but also prevents the generation of these highly toxic radicals^17^. When control rats were exposed to 95 dB SPL octave band noise, 8 h/d for 21 days, they developed moderate, mid-frequency hearing loss and moderate outer hair cell (OHC) loss^18^. However, if noise-exposed rats were treated with HK-2 before, during and after the exposure, both the hearing loss and hair cell loss were significantly reduced in a dose-dependent manner.

Given these encouraging results against moderately traumatic noise, a logical next step was to determine if HK-2 could protect against hearing impairments caused by more damaging acoustic blast waves. Because blast-induced hearing damage has been reported to stem mainly from direct mechanical damage to the cochlea rather than oxidative stress^19^, therapies designed to reduce oxidative stress might prove ineffective against blast-induced mechanical damage to the cochlea. However, antioxidant therapy might protect cochlear cells located on the penumbra of the mechanical lesion where the organ of Corti remains intact. This premise is supported by a report that antioxidant therapy protected against a blast wave exposure that caused moderate hearing loss and hair cell loss^20^.

Intense blast wave exposures can cause dizziness and balance problems, conditions associated with damage to the peripheral vestibular system. Hair cells in the otolithic balance organs, the saccule and utricle, are believed to be more vulnerable to blast wave exposure than hair cells located in the ampullae of the semicircular canals^21^. However, persistent damage to hair cell stereocilia has been observed in the cristae in the semicircular canals as well as in the utricle and saccule. Vestibular hair cell damage has been linked to behavioral vestibular deficits and abnormal vestibular-evoked eye movements^22^. Some reports suggest that vestibular disorders and vestibulotoxicity result from elevated levels of oxidative stress^23–25^. This raises the possibility that HK-2 might ameliorate blast-induced damage to vestibular hair cells.

Based on our earlier reports and those of others, anatomical studies were conducted to determine if HK-2 could reduce blast wave induced injury to the cochlea and peripheral vestibular system. We exposed four groups of rats to six blast waves at 5 min intervals (186 dB peak SPL) and compared the amount of cochlear and vestibular hair cell loss and the degree of cochlear neuronal damage in a (1) blast exposed group (B), (2) a blast exposed group treated with earplugs (BEP), (3) a blast exposed group treated with HK-2 (BHK-2) and (4) a blast exposed group treated with both earplugs and HK-2 (BEPHK-2). Technical limitations prevented the collection of functional data to assess the efficacy of the treatments at various times following the blast exposure.

## Results

### Outer hair cells

**Figure 1:**
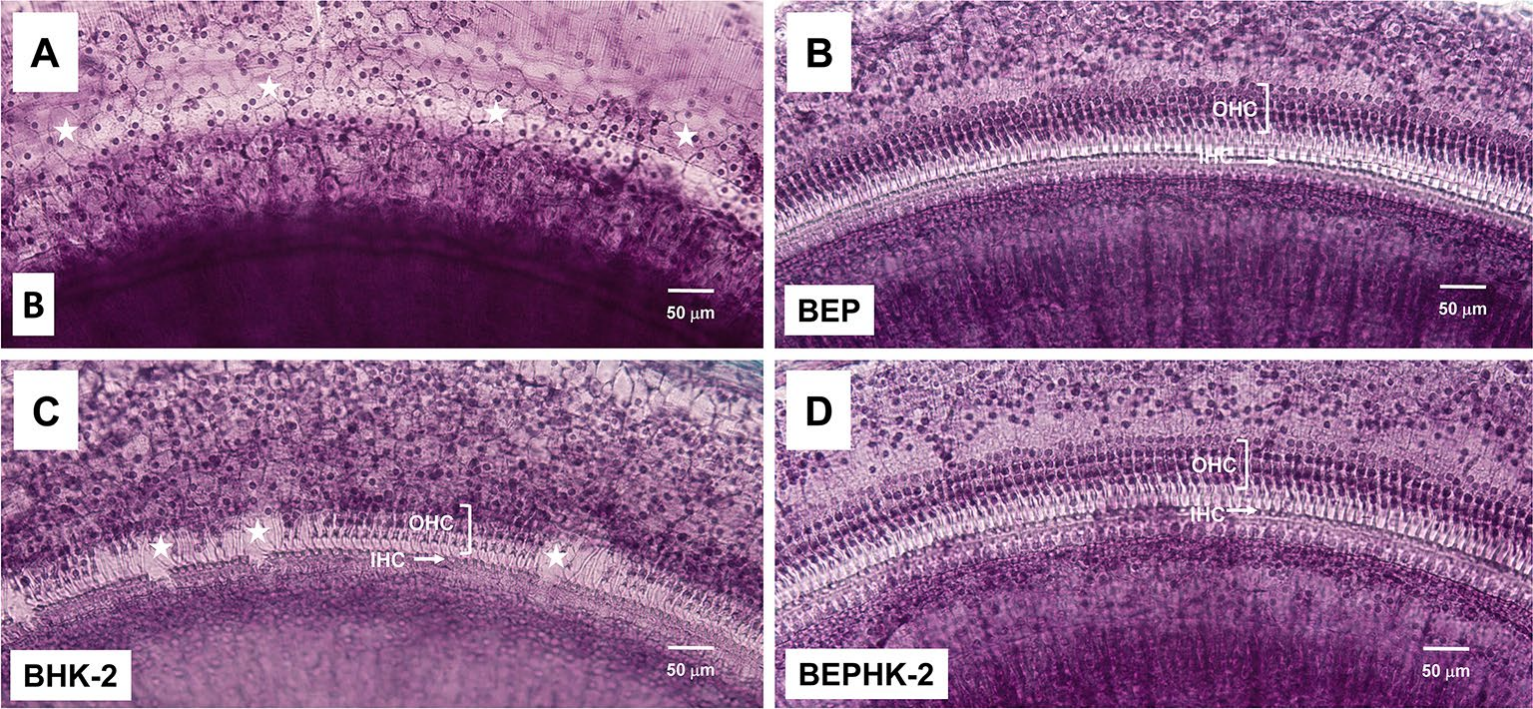
HK-2 suppresses blast-induced hair cell loss. Representative photomicrograph of surface preparations from the middle of basal turn of the cochlea stained with Harris hematoxylin. Cochlea evaluated 2-months after exposure to 6 blasts of ~ 186 dB peak sound pressure level. Results from (**A**) blast-alone (**B**) group, (**B**) blast group with earplugs (BEP), (**C**) blast group treated with HK-2 (BHK-2) and (**D**) blast group treated with earplugs and HK-2 (BEPHK-2). In the B group, nearly all outer hair cells (OHC) and inner hair cells (IHC) are missing and replaced by large flat, cuboidal cells (panel A, white stars). Nearly all OHC and IHC present in blasted-exposed rats treated with ear plugs (**B**) or earplugs combined with HK-2 (D). Surface preparations from blast-exposed rats treated with HK-2 (**C**) shows patches of missing hair cells and flattened epithelium (white stars) interspersed with regions where rows of OHC and IHC are present.

To determine if HK-2 could protect cochlear hair cells from six blast exposures (186 dB peak SPL, once every 5-min), one cochlea from each of the rats in the four groups was evaluated along the entire length of the basilar membrane. The blast exposure damaged a large portion of the organ of Corti in the unprotected blast wave group. Figure 1A shows a representative photomicrograph of a surface preparation taken from the middle of the basal turn of the cochlea of an untreated rat in the B group. Because the blast exposure destroyed all of the hair cells and support cells, the missing organ of Corti was replaced by a flattened epithelium comprised largely of cuboidal-shaped cells with a nucleus surrounded by pale cytoplasm (Fig. 1A, red star). In blast-exposed rats fitted with earplugs (BEP), the hair cells and support cells remained largely intact as illustrated by the surface preparation of the organ of Corti in the middle of the basal turn (Fig. 1B). Three parallel rows of OHC and a single row of IHC were present along the length of the organ of Corti. Among the rats treated only with HK-2 in the BHK-2 group, patches of OHC and IHC were missing along the organ of Corti in the middle of the basal turn (Fig. 1C); damage was more extensive to OHC than IHC. Among the rats in the BEPHK-2 group fitted with earplugs and treated with HK-2, there was little or no evidence of OHC or IHC loss in the middle of the basal turn (Fig. 1D).

**Figure 2:**
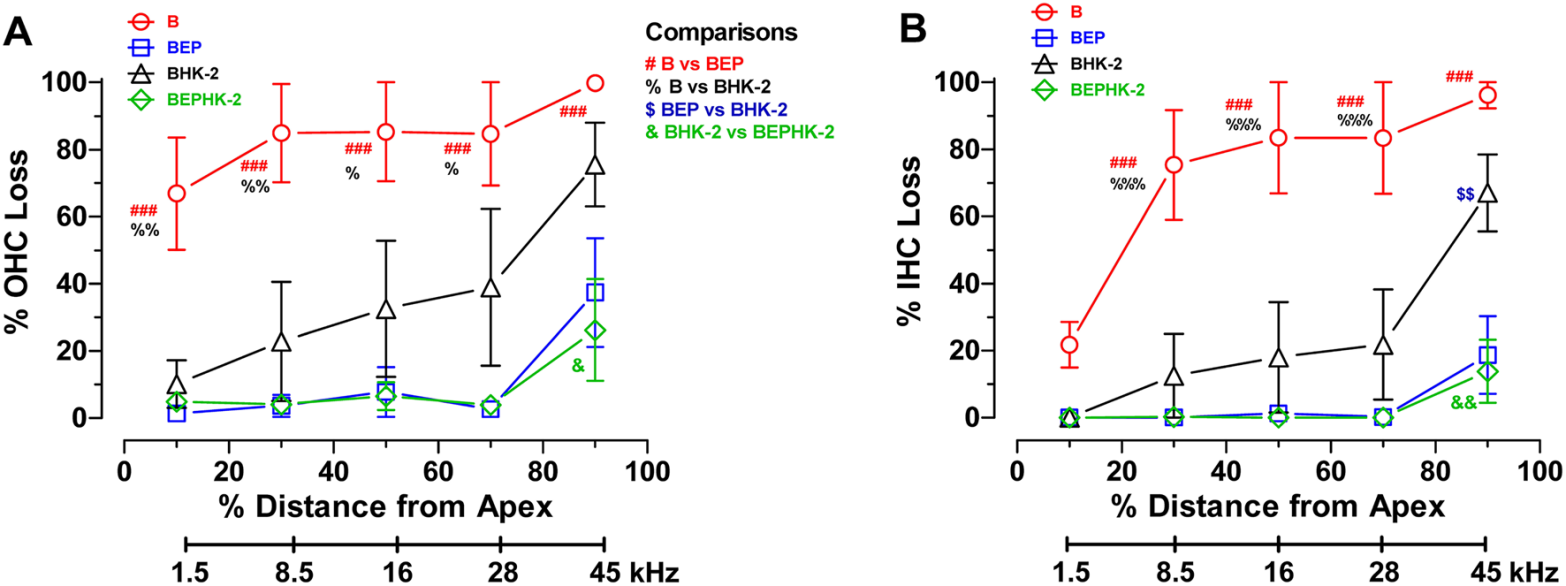
HK-2 prevents blast-induced loss of outer hair cells (OHC) and inner hair cells (IHC). Mean (n = 6, + /− SEM) cochleograms for the B, BEP, BHK-2 and BEPHK-2 groups. Cochleograms show percent (**A**) OHC loss and (**B**) IHC loss versus percent distance from the apex of the cochlea (20% intervals). Lower bar shows rat tonotopic map^26^. A two-way repeated measures analysis revealed a significant effect of treatment (*p* < 0.0001) and cochlear location (*p* < 0.0001); see text for details. Bonferroni post-test test revealed significant differences in the amount of OHC or IHC loss between B versus BEP group (#), B versus BHK-2 group (%), BEP versus BHK-2 group ($) and BHK-2 and BEPHK-2 group (&). 1, 2 and 3 symbols correspond to *p* < 0.05, 0.01 and 0.001; see text for details.

To quantify the results, cochleograms were prepared from one cochlea of each animal in each of the four groups. Mean percent OHC and IHC loss were determined over 20% intervals along the cochlea for each rat. These data were used to construct mean cochleograms (n = 6/group, + /− SEM) for each group showing the percent OHC loss in 20% intervals from the apex to the base of the cochlea. Cochlear location was related to frequency using a rat tonotopic map^26^. Figure 2A compares the mean OHC losses in the four groups. Mean OHC losses in the B group declined from ~ 100% near the high-frequency base of the cochlea to ~ 67% in the low-frequency apex. Mean OHC losses in the BEP group were substantially less than in the B group; OHC losses in the BEP group decreased from ~ 37% near the base of the cochlea to less than 9% near the apex. OHC losses in the BHK-2 group were also substantially less than in the B group. BHK-2 OHC loss was ~ 76% in the high-frequency base of the cochlea, declining to ~ 39% and then 10% at the 70% and 10% locations respectively. OHC losses in the BEPHK-2 group were nearly identical to those in the BEP group except in the extreme base where the loss was about 10% less in the BEPHK-2 group. A two-way repeated measures analysis of variance of OHC loss revealed a significant treatment effect (F_3, 80_ = 14.71, *p* < 0.0001) and a significant effect of cochlear location (F_4, 80_ = 14.57, *p* < 0.0001). Bonferroni post-testing revealed significant differences in OHC loss between the B versus BEP groups (ear plug protective effect) at all cochlear locations (*p* < 0.001); significant differences in OHC loss between the B versus BHK-2 groups (HK-2 protective effect) at cochlear locations of 10, 30, 70% (*p* < 0.01) and 50% (*p* < 0.05), and a significant difference in OHC loss between the BHK-2 versus BEPHK-2 groups at the 90% cochlear location (*p* < 0.05).

### Inner hair cells

The blast wave exposure caused extensive IHC damage. Mean IHC loss in the B group decreased from ~ 96% near the base of the cochlea, decreasing to ~ 80% near the middle of the cochlea and then declining to ~ 22% near the apex (Fig. 2B). IHC losses in the BEP group and BEPHK-2 group were minimal except for losses ~ 14–19% in base of the cochlea. Mean IHC losses in the BHK-2 were much less than in the B group particularly in the apical two-thirds of the cochlea. Mean IHC loss in the BHK-2 group was ~ 67% near the base of the cochlea, declining to ~ 20 in the middle of the cochlea and then falling to 0% near the apex. A two-way repeated measures analysis of variance revealed a significant treatment effect (F_3, 80_ = 16.35, *p* < 0.0001) and a significant effect of cochlear location (F_4, 80_ = 23.63, *p* < 0.0001). Bonferroni post-testing revealed significant differences in IHC loss between the B versus BEP groups (ear plug protective effect) at 30%, 50%, 70% and 90% cochlear locations (*p* < 0.001); significant differences in IHC loss between the B versus BHK-2 groups (HK-2 protective effect) at cochlear locations of 30, 50 and 70% (*p* < 0.001), a significant difference in IHC loss between BEP and BHK-2 groups at the 90% location (*p* < 0.01) and a significant difference in IHC loss between the BHK-2 versus BEPHK-2 group at the 90% cochlear location (*p* < 0.01).

### Auditory nerve fibers

**Figure 3:**
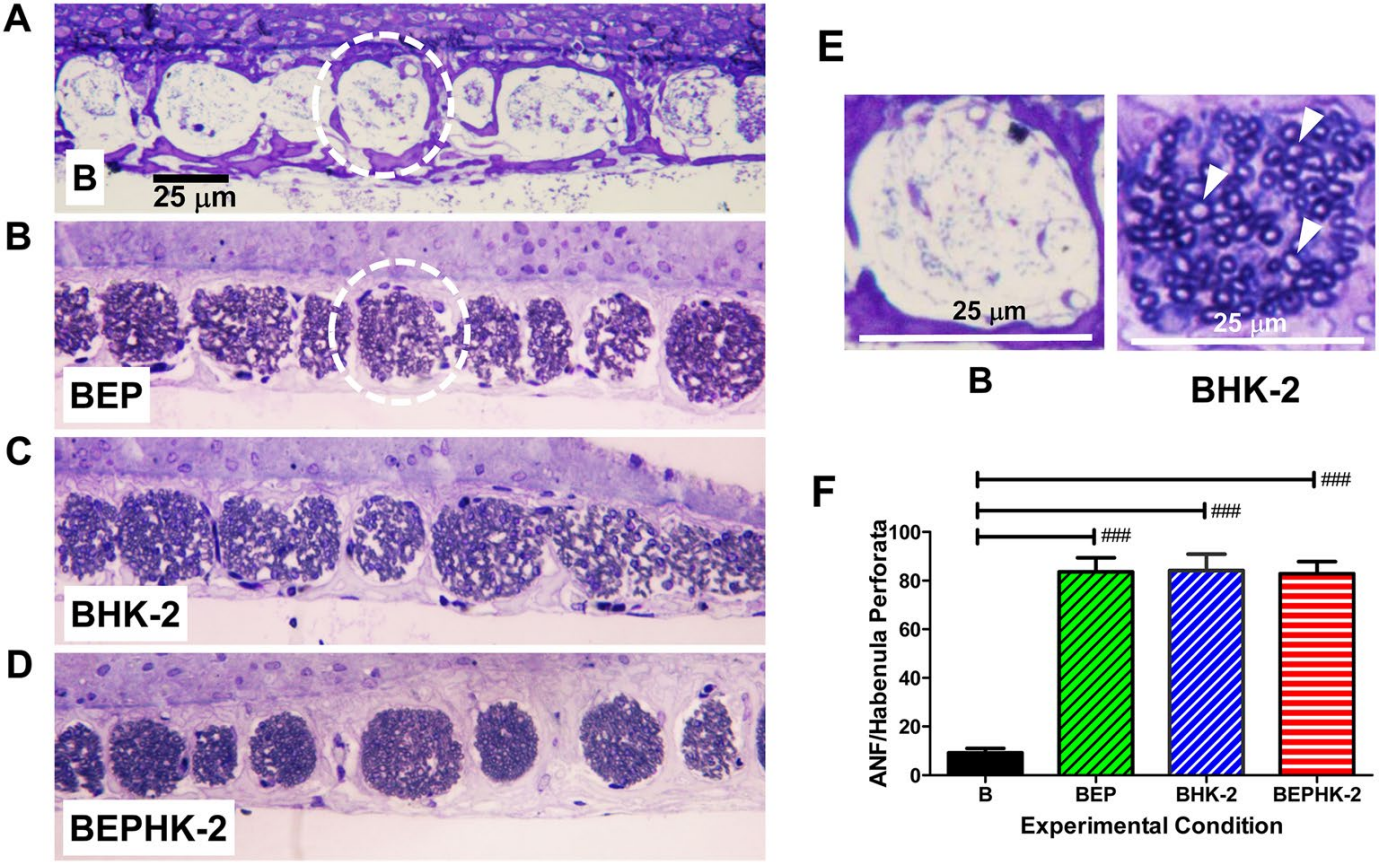
HK-2 suppresses blast-induced loss of auditory nerve fibers (ANF). Representative photomicrographs of toluidine blue stained, 3 µm sections from middle of basal turn of cochlea. Data obtained 2-months after exposure to 6 blasts of ~ 186 dB peak sound pressure level. Results show habenula perforata in (**A**) B group, dashed circle, (**B**) BEP group, (**C**) BHK-2 group, and (**D**) BEPHK-2 group. (**E**) High magnification view of habenula perforata in B group devoid of ANF versus BHK-2 group filled with toluidine blue stained ANF. ANF characterized by darkly stained ring of membrane surrounding pale cytoplasm (arrowheads). (**F**) Mean (n = 6, + /− SEM) number of ANF per habenula perforata in B group significantly less (p < 0.001) than in BEP group, BHK-2 group and BEPHK-2 group; see text for details.

Large IHC losses are often accompanied by ANF degeneration^27^. To quantify the degree of ANF degeneration, we counted the number of ANF in the habenula perforata in the middle of the basal turn of the cochlea (~ 25 kHz location). A massive loss of ANF was evident in the habenula perforata of rats in the B group as illustrated by the representative photomicrograph in Fig. 3A. In contrast, the habenula perforata was filled with ANF in the BEP (Fig. 3B), BHK-2 (Fig. 3C) and BEPHK-2 (Fig. 3D) groups. The left panel of Fig. 3E show a high magnification view of the habenula perforata from a rat in the B group largely devoid of ANF. In contrast, the right panel of Fig. 3E shows a high magnification view of the habenula perforata from a rat in the BHK-2 group. The ANF in the habenula perforata of rats in the BHK-2 group were characterized by a darkly stained ring of membrane surrounding a lightly-stained cytoplasmic interior (arrowheads). Counts were made of the number of ANF in each habenula perforata (see Methods), the mean number of ANF was determined for each rat and the data used to compute the mean number of ANF per habenula perforata (n = 6, + /− SEM) for each group. The mean number of ANF/habenula perforata was ~ 9.2 in the blast alone group (B) versus 83.7, 84.2 and 82.8 in the BEP, BHK-2 and BEPHK-2 groups respectively. There was a significant difference across groups (one-way analysis of variance, F_3, 20_ = 52.45, *p* < 0.0001). The number of ANF/habenula perforata in the B group was significantly less (Newman-Keuls Multiple comparison) than in the BEP (*p* < 0.05), BHK-2 group (*p* < 0.05) and BEPHK-2 group (*p* < 0.05). There were no significant differences among the BEP, BHK-2 and BEPHK-2 groups. Thus, HK-2 provided complete protection against blast-induced ANF degeneration, protection equal to BEP and BEPHK2.

### Spiral ganglion neurons

**Figure 4:**
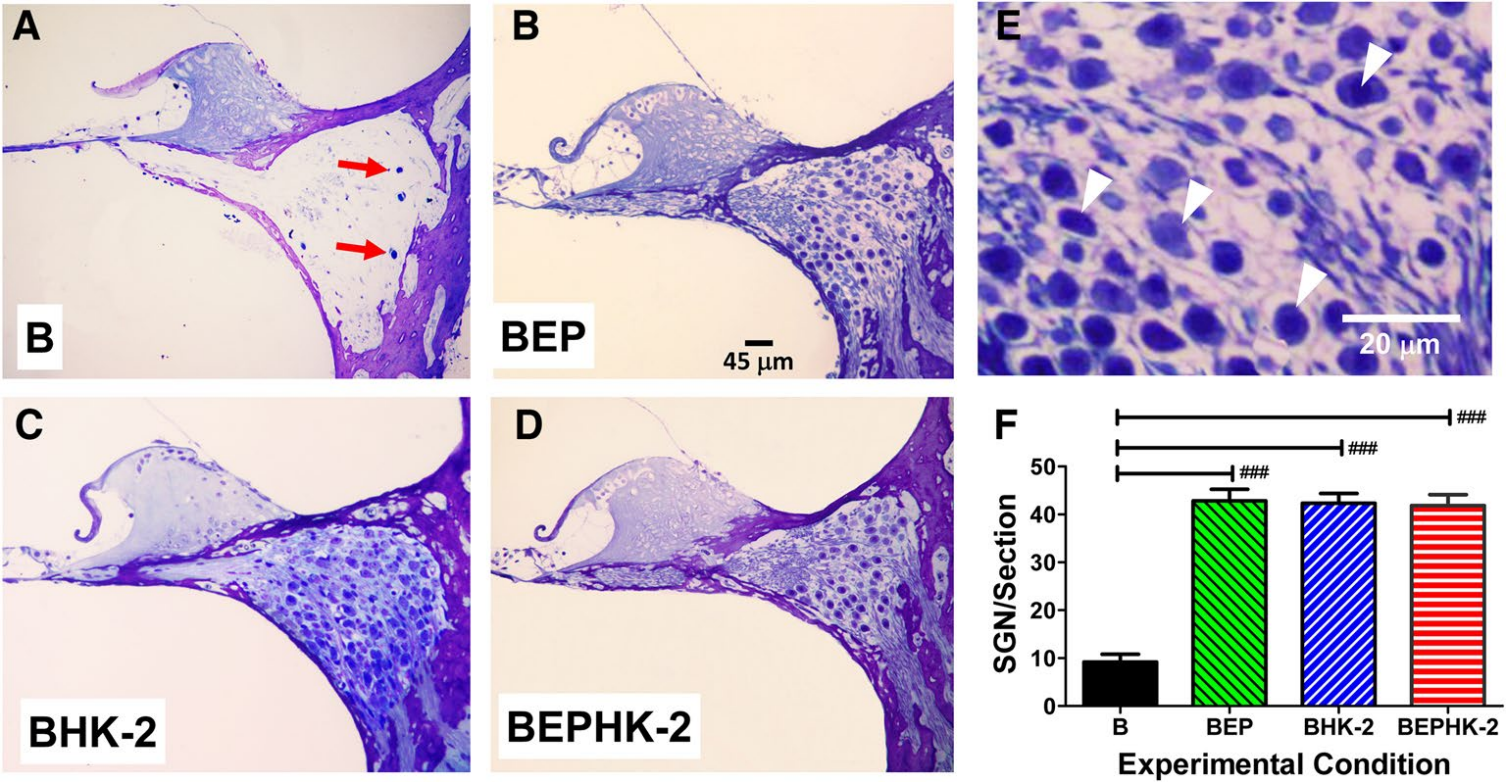
HK-2 attenuates blast-induced degeneration of spiral ganglion neurons (SGN) in Rosenthal’s canal. Representative photomicrograph of toluidine blue stained sections from the middle of the basal turn of cochlea (25 kHz location) 2-months after exposure to 6 blasts of ~ 186 dB peak sound pressure level. Sections from (**A**) B group, (**B**) BEP group, (**C**) BHK-2 group, and (**D**) BEPHK-2 group. Red arrows in Fig. 4A point to a few surviving SGN in Rosenthal’s canal. (**E**) High magnification view of toluidine blue stained SGN soma (arrowheads). (**F**) Mean (n = 6, + /− SEM) number of SGN per section in B group significantly less (###, *p* < 0.001) than in BEP group, BHK-2 group and BEPHK-2 group; see text for details.

The noise-induced destruction of many IHC generally results in the loss of SGN. To determine the extent of blast-induced neural degeneration, we counted the number of SGN in radial sections from the middle of the basal turn of the cochlea (~ 25 kHz location). Figure 4A–D show representative cross sections of Rosenthal’s canal from rats in the experimental groups. Rosenthal’s canal in the B group was largely devoid of SGN except for a few lone survivors (Fig. 4A). In contrast, Rosenthal’s canal in the BEP group (Fig. 4B), BHK-2 group (Fig. 4C) and BEPHK-2 group (Fig. 4D) were filled with SGN. Figure 4E shows a high magnification view of Rosenthal’s canal from a rat in the BEP group. SGN were characterized by a darkly stained nucleus surrounded by lightly stained cytoplasm (arrowheads). Counts were made of the number of SGN in each cross section (see Methods for details) and the mean number of SGN per section was determined for each cochlea. The individual data were used to determine the mean (n = 6, + /− SEM) number of SGN/section in each group. The mean number of SGN per section was ~ 9 in the B group versus 42.8, 42.3 and 41.8 in the BEP, BHK-2 and BEPHK-2 groups respectively. There was a significant difference across groups (one-way analysis of variance, F_3, 20_ = 62.32, *p* < 0.0001). The number of SGN/section in the B group was significantly less (Newman-Keuls Multiple comparison) than in the BEP group (*p* < 0.05), BHK-2 group (*p* < 0.05) and BEPHK-2 group (*p* < 0.05); there were no significant differences among these three groups. These results show that HK-2 provided significant protection against blast-induced SGN degeneration, protection equal to EP alone and EP + HK2.

### Saccule hair cell densities in striola and marginal regions

**Figure 5:**
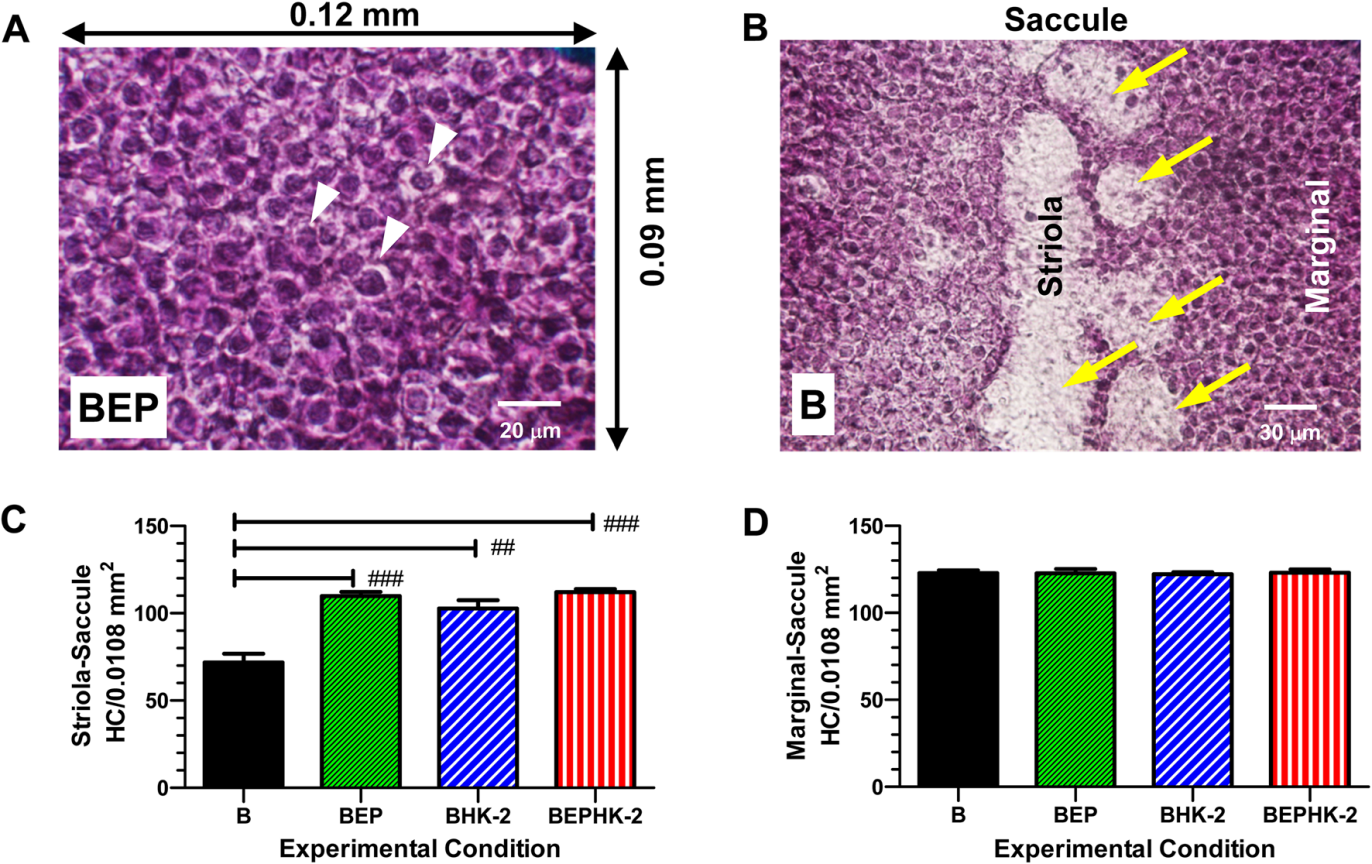
HK-2 reduces blast-induced degeneration of vestibular hair cells in saccule. (**A,B**) Representative photomicrographs of surface preparations of saccule in vicinity of striola; specimens stained with Harris hematoxylin. (**A**) Representative photomicrograph of saccule obtained from rat in the BEP group two months post-blast. White arrowheads point to darkly stained nuclei of vestibular hair cells surrounded by lightly stained nerve terminals. Number of vestibular hair cells counted in 0.12 × 0.09 mm areas (0.0108 mm^2^) in striola and marginal regions of saccule. (**B**) Representative photomicrograph from saccule of rat in blast exposed group (**B**) two months post-exposure. Many hair cells missing (yellow arrows) from center of striola and in adjacent circular patches in contrast to high density of vestibular hair cells in marginal area. (**C**) Mean (n = 6, + /− SEM) hair cell density in striola region of saccule. Hair cell density in the B group significantly less than in the BEP group (*p* < 0.001), BEPHK-2 group (*p* < 0.001) and BHK-2 group (*p* < 0.01). (**D**) Mean (n = 6, + /− SEM) hair cell density in marginal region of saccule. Hair cell densities in the four groups (B, BEP, BEPHK-2 and BHK-2) were similar and not significantly different from one another.

Because blast wave exposures reportedly damage vestibular hair cells^21,22^, we measured hair cell densities in the macula of the saccule, utricle and the crista ampullae of the three semicircular canals. Figure 5A shows a representative photomicrograph of a surface preparation of the maculae of the saccule from a rat in the BEP group. Vestibular hair cells in the BEP group were densely packed throughout the epithelium and were characterized by a darkly-stained nucleus surrounded by pale cytoplasm. In the striola region of the saccule of the B group (Fig. 5B), vestibular hair cell density was greatly reduced in a large stripe surrounded by circular patches of missing hair cells. In contrast, vestibular hair cell density in the surrounding marginal region of the saccule appeared normal.

Vestibular hair cell densities were measured in the striola and marginal regions of the saccule of each animal (see details in Methods) and mean (n = 6, + /− SEM) hair cell densities were computed for each group. Mean vestibular hair cell densities in the striola of the BEP, BHK-2 and BEPHK-2 groups were 109.8, 102.7 and 112.0 hair cell/0.0108 mm^2^ respectively versus 71.83 hair cells/0.0108 mm^2^ in the B group, about 35% less than the other three groups. A one-way analysis of variance revealed a significant difference in hair cells density across groups (F_3, 20_ = 24.35, *p* < 0.0001). Bonferroni post-testing indicated that striola hair cell density in the B group was significantly less than in the BEP (*p* < 0.05), BHK-2 (*p* < 0.05) and BEPHK-2 groups (*p* < 0.05). However, striola hair cell densities were not significantly different from one another in the BEP, BHK-2 and BEPHK-2 groups. Figure 5D shows the mean (n = 6, + /− SEM) hair cell densities in the marginal region of the saccule for the four experimental groups. Mean hair cell densities in the marginal cell region of the saccule were 122.8, 122.7, 122.2 and 123.0/0.0108 mm^2^ for the B, BEP, BHK-2 and BEPHK-2 group respectively. A one-way analysis of variance revealed no significant difference in marginal hair cell densities across the four groups (F_3, 20_ = 0.037, *p* = 0.9901). Thus, HK-2 provided significant protection against blast-induced hair cell loss in the striola of the saccule; a protective effect equivalent to that provided by earplugs alone.

### Utricle and ampullae hair cell densities

**Figure 6:**
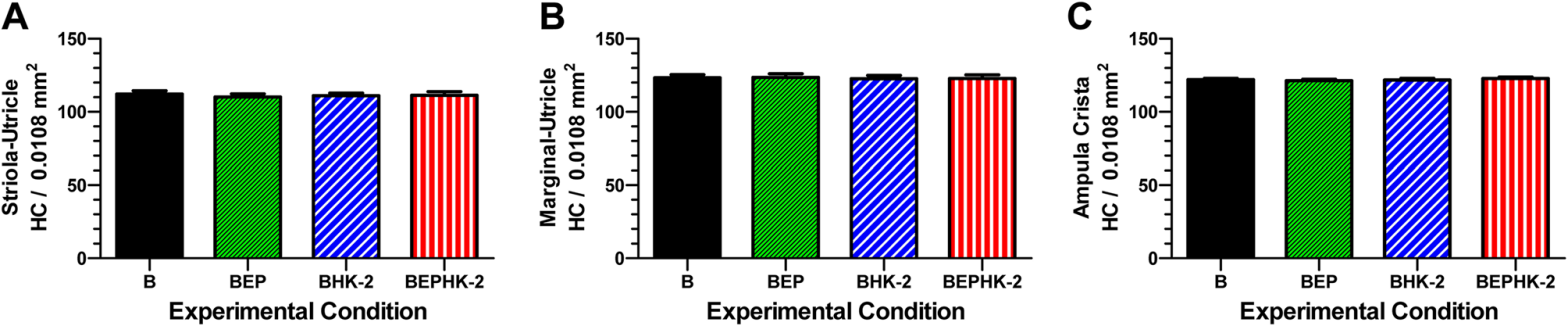
Blast wave exposure did not alter hair cell densities in the striola and marginal regions of the utricle and the ampulla of three semicircular canals. (**A**) Mean (n = 6, + /− SEM) vestibular hair cell densities (HC/0.0108 mm^2^) in the striola region of the utricle; note nearly identical values across B, BEP, BHK-2 and BEPHK-2 experimental groups. (**B**) (**A**) Mean (n = 6, + /− SEM) vestibular hair cell densities (HC/0.0108 mm^2^) in the marginal region of the utricle nearly identical across B, BEP, BHK-2 and BEPHK-2 experimental conditions. (**C**) Mean (n = 18, + /− SEM) vestibular hair cell densities (HC/0.0108 mm2) in ampulla of crista of three semicircular canals (n = 6/canal) nearly identical across B, BEP, BHK-2 and BEPHK-2 experimental conditions.

Hair cell densities were also evaluated in the macula of the utricle and cristae ampullae of the three semicircular canals. There was no apparent loss of hair cells in the macula of the utricle. Mean hair cell densities in the striola region of the macula of the utricle were 112.2, 110.2, 111.0 and 111.3/0.0108 mm^2^ for the B, BEP, BHK-2 and BEPHK-2 groups respectively (Fig. 6A, n = 6/group, + /− SEM), values similar to those seen in normal controls. There was no significant difference between groups (one-way analysis of variance, F_3, 20_ = 0.146, *p* = 0.931). Mean hair cell densities in the marginal region of the macula of the utricle were 123.3, 123.5, 122.7 and 122.8/0.0108 mm^2^ for the B, BEP, BHK-2 and BEPHK-2 groups respectively (Fig. 6B, n = 6/group, + /-SEM), values within the normal range. There was no significant difference between groups (one-way analysis of variance, F_3, 20_ = 0.028, *p* = 0.993). Mean hair cell densities in the crista of the ampulla of the three semicircular canals were 122, 121.3, 121.8 and 122.8/0.0108 mm^2^ for the B, BEP, BHK-2 and BEPHK-2 groups respectively (Fig. 6C, n = 18, 6/experimental group, + /− SEM). There were no significant difference between groups (one-way analysis of variance, F_3, 20_ = 0.3626, *p* = 0.78). Taken together, these results indicate that blast wave induced vestibular hair cell loss was confined to the striola region of the saccule.

## Discussion

Exposure to intense blasts can ostensibly cause direct mechanical damage to cochlear hair cells, support cells and auditory nerve fibers that synapse on the hair cells^19,28,29^. These observations suggested that HK-2, a multifunctional redox modulator, might provide little or no protection against blast induced-mechanical damage to cochlear hair cells and neurons. However, blast-induced hearing loss likely involves multiple mechanisms such as neuroinflammation, excitotoxicity, toxic mixing of endolymph and perilymph, and disruption of the blood-labyrinth barrier, factors likely to contribute to oxidative stress^29–31^. Consistent with this view, combination antioxidant therapy was previously shown to greatly reduce temporary and permanent blast-induced hearing loss and OHC loss suggesting that the therapy protected against both the mechanical and metabolic aspects of the exposure^20,32^. These results suggest that antioxidant therapies may be effective at preventing hearing loss and cochlear damage from both blast wave and steady-state noise exposure^33^.

### Cochlear hair cell loss

Our results provide compelling evidence that HK-2, a MFRM, provides significant protection against blast-induced destruction of OHC, IHC, ANF and SGN. Treatment with HK-2 reduced total OHC loss by ~ 67% and total IHC loss by ~ 57.3%. HK-2 was least effective in preventing OHC and IHC loss in the most basal part of the cochlea where the blast-induced lesion was nearly 100% (Fig. 2A). The massive loss of OHC and IHC in the extreme base of the cochlea was likely the result of immediate and direct mechanical destruction of the sensory epithelium as described previously^19^. However, HK-2 provided significant protection of OHC and IHC in the apical three-fourths of the cochlea where OHC and IHC losses were 80% or less (Fig. 2A,B). Earplugs provided nearly complete protection against blast-induced OHC and IHC loss except in the most basal segment of the cochlea where ~ 40% of OHC and ~ 20% of IHC were missing. The OHC and IHC lesions in this region are most likely caused by direct mechanical destruction of the organ of Corti because combining HK-2 with earplugs did not significantly reduce the hair cells lesions compared to earplugs alone (blue squares versus green diamond symbols, Fig. 2A,B).

**Figure 7:**
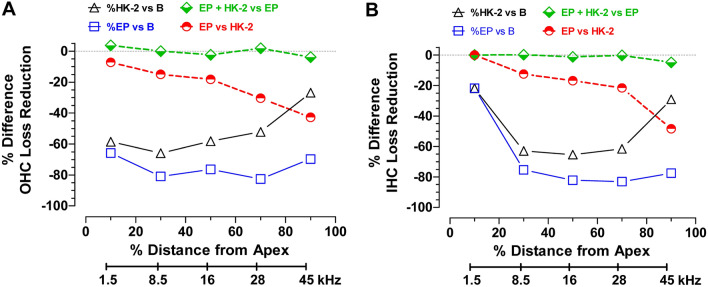
Earplugs (EP) provide moderately greater protection against blast-induced hair cell loss than HK-2; HK-2 combined with EP failed to provide additional protection. Four comparisons showing the percent difference in reduction of (**A**) OHC loss and (**B**) IHC loss as function of cochlear location/frequency: (a) EP vs B: EP provided the greatest reduction of blast-induced OHC and IHC loss over most of the cochlea; reduction of hair cell loss nearly constant across cochlear location/frequency except for greatly reduced IHC loss near the apex of the cochlea. (b) HK-2 vs B: HK-2 provided the second greatest reduction of OHC and IHC losses. Reduction of OHC loss greatest near low-frequency apex declining toward high-frequency base. IHC loss reductions greatest in middle/mid-frequency region of cochlea. (c) EP vs HK-2: EP provided greater reductions in OHC and IHC losses than HK-2; EP protective effect increased from low-frequency apex towards high-frequency base. (d) EP + HK-2 vs EP: No further reduction of OHC and IHC loss for EP plus HK-2 versus EP alone (i.e., lack of synergy).

To aid in the interpretation of the data, Fig. 7 compares the percentages by which OHC (panel A) and IHC (panel B) losses were reduced as function of cochlear location for four conditions. The EP vs B comparison shows that EP reduced OHC losses by 65–80% (Fig. 7A) and IHC losses (Fig. 7B) by ~ 80% over most of the cochlea except in base where OHC and IHC loss reductions were ~ 28% and ~ 20% respectively. The HK-2 vs B comparison shows that HK-2 reduced OHC losses ~ 55–65% over most of the cochlea except near the base where the reduction was ~ 28% (Fig. 7A). HK-2 (Fig. 7B) reduced IHC losses ~ 65% in the middle of the cochlea, but reductions declined to ~ 30% near the base and ~ 22% near the apex of the cochlea. The EP vs HK-2 comparison shows that EP provided greater protection than HK-2 (Fig. 7A,B); OHC and IHC loss reductions for EP exceeded those of HK-2 by ~ 45% in the base, 20% in the middle and approached zero near the apex of the cochlea. The EP + HK-2 vs EP comparison showed no meaningful interaction between the mechanical protection provided by EP and the biochemical protection provided by HK-2. From these results, we propose that the red-dashed line in Fig. 7 (EP vs HK-2) represents the tonotopic weighted average of the direct mechanical damage to the cochlea that cannot be prevented by the biochemical actions of HK-2; the magnitude of this mechanical damage is greatest near the base and declines towards the apex of the cochlea. We hypothesize that the area between the HK-2 vs B curve and EP vs HK-2 curve represents the amount by which the biochemical action of HK-2 protected against hair cell loss. The HK-2 biochemical rescue of hair cells is greatest in the middle and apical regions of the cochlea while its biological rescue of hair cells is negligible near the base of the cochlea where the HK-2 vs B curve and EP vs HK-2 curves intersects and where mechanical damage is greatest.

HK-2 was not as effective as earplugs at preventing blast-induced OHC or IHC loss. If this difference is due to metabolic rather than mechanical factors, then we can speculate on possible reasons for this. One possibility is that the dose of HK-2 used in this study was too low to completely eliminate all blast-induced oxidative stress. In our previous NIHL study, HK-2, administered at 40 mg/kg/d for 10 days post-exposure, provided significant protection reducing OHC loss by ~ 25%^18^. In an attempt to increase its efficacy, HK-2 was administered at 50 mg/kg/d for 30 days post-blast. This dose, which was 25% greater and administered three times longer than that used previously, reduced OHC loss by as much at 60–65% (Fig. 7), raising the possibility of greater efficacy with higher HK-2 doses and/or longer treatment durations. This hypothesis could be tested by conducting a dose–response study to identify the most effective dose. An alternative explanation is that oxidative stress is one of several biological mechanisms that contributes to blast-induced hair cell death. Many other factors such as neuroinflammation, excitotoxicity, and neurovascular impairment could be involved in NIHL and other therapeutic interventions alone or in combination with HK-2 could be tested to assess their efficacy^28,34–39^.

### Auditory nerve fibers and spiral ganglion

Our analysis focused on ANF and SGN degeneration in the middle of the basal turn of the cochlea near the 25 kHz region of the cochlea. The blast exposure caused a massive loss of ANF (Fig. 3F) and SGN (Fig. 4F) relative to the earplug group (BEP). Approximately 89% of the ANF and 79% of the SGN were destroyed by the blast exposure (re the EP group). Treatment with HK-2 restored ANF counts to essentially normal levels (~ eightfold increase) and SGN counts to normal values (~ 3.7 fold increase). Thus, HK-2 provided complete protection of ANF and SGN in the 25 kHz region of the cochlea whereas the drug provided only partial protection of OHC and IHC in the same region. While the evidence for “complete” protection of ANF and SGN is impressive, these results should be interpreted cautiously because our analyses were only carried out in the 25 kHz region of the cochlea. Additional studies are need to determine the extent to which HK-2 protects ANF and SGN in more apical and basal regions of the cochlea. Furthermore, ANF and SGN degeneration can occur over many months or years depending on the species^40^. Therefore, it would be important to assess the survival of ANF and SGN at much longer survival times to determine if HK-2 can protect against long-term neural degeneration. Prior noise exposure has also been reported to accelerate age-related hearing loss suggesting the need to assess SGN and ANF losses at much longer survival times^41^. Oxidative stress remains elevated in the organ of Corti for at least 14 day following exposure to intense continuous noise^16^. However, it is possible that blast-induced oxidative stress remains elevated for a much longer time given the massive amount of cochlear damage that occurred. Massive damage could prolong the period of SGN and ANF degeneration. To determine if a prolonged period of SGN and ANF degeneration is occurring, cochlear tissues would need to be harvested at post-blast survival times both shorter and longer than the 60 day survival time employed in this study.

Some NIHL studies suggest that IHC are required for SGN survival; however, more recent findings in genetic mutants demonstrate that SGN can survive in the absence of IHC^42^. The authors suggested that SGN loss following noise exposure may be caused by delayed metabolic secondary damage to ANF or SGN. In so, then HK-2 would appear to have neuroprotective effects, consistent with previous studies showing that HK-2 is neuroprotective in other systems^17^. These results suggest a novel use of HK-2, namely preventing the degeneration of ANF and SGN in cochlear implant patients^43,44^. One important question that remains to be answered is how long the neuroprotective effects of HK-2 lasts. We administered HK-2 for 30 days following the blast exposure and harvested the tissues 60 days post-exposure. If the ANF and SGN were to degenerate at much longer survival times, this would suggest that there is a prolonged period of oxidative stress following the blast exposure. If no further degeneration occurred at much longer survival time, then one might conclude that a heightened period of oxidative stress mainly occurs 30 days or less following the blast exposure. Future experiments in which the duration or timing of HK-2 treatment is varied would help to resolve these questions.

### HK-2 protects saccular hair cells in the striola

Some studies suggest that blast exposures damage the stereocilia on vestibular hair cells while others have failed to observe vestibular hair cell loss^45^. Our results clearly show that six blasts of 186 dB pSPL selectively destroyed hair cells in the macula of the saccule, but only in the striola region (Fig. 5B,C), but not the marginal zone (Fig. 5B,D). There was no evidence of hair cell loss in either the striola or marginal zones of the macula of the utricle (Fig. 6A,B) and no evidence of hair cell loss in the crista of the ampulla in the three semicircular canals. The enhanced vulnerability of saccular hair cells in striola region relative to hair cells in the utricle and semicircular canals is likely related to its closer proximity to the stapes which transmits pressure fluctuation not only to the cochlea, but also the saccule, consistent with the observation that saccular afferent nerve fibers respond to sound^46^. It is unclear from our results why hair cells in the striola region of the macula of the saccule are more susceptible to blast trauma than those in the marginal region. Sensitivity to blast trauma could be related to regional differences in stereocilia bundle length, stiffness, and sensitivity and the magnitude of the forces applied to the hair cell lining the macula^47,48^. The blast exposure destroyed approximately one-third of the hair cells in the striola region of the saccule. Importantly, HK-2 treatment restored hair cell densities to normal levels similar to those measured in the BEP and BEPHK-2 groups.

### Limitations

One limitation of the current study is the lack of physiological measures such as distortion product otoacoustic emissions (DPOAE) or auditory brainstem response (ABR) to assess the functional status of the OHC and neural output of the cochlea and central nervous system following the blast exposure^49–51^. It would be interesting to compare how DPOAE and ABR amplitudes change in the treatment groups over the 60 day recovery period. Is the recovery process more prolonged in the BHK-2 group compared to the BEP group? How well do the DPOAE amplitudes correlate with the magnitude of the OHC lesions in each of the treatment group? HK-2 might enhance OHC survival, but damage to OHC stereocilia might prevent the recovery of DPOAE amplitudes. Is the amplitude of wave I or wave V of the ABR correlated with ANF and SGN losses in each group? EP and HK-2 might enhance ANF and SGN survival, but ABR responses might be weak or absent because of damage to the IHC- ANF synapse. Another shortcoming is the use of a single dose/duration of HK-2 treatment. Would a higher dose or longer duration of HK-2 treatment further enhance hair cells survival? Blast wave exposures can damage the central nervous system resulting in cognitive impairment and suppression of neurogenesis in the hippocampus^52,53^. Does HK-2 treatment prevent these impairments? The blast wave exposure destroyed vestibular hair cells in the striola region of the saccule and EP and HK-2 prevented this damage. Future studies would benefit from functional tests such as the vestibular evoked myogenic potential to determine if the hair cell lesions in the striola region of the saccule leads to deficits that can be prevented by HK-2 or EP^54–56^. HK-2 prevented the loss of ANF and SGN in the middle of the basal turn two months following the blast wave exposure. However, it is unclear if HK-2 provides a similar degree of protection in more apical or basal regions of the cochlea and whether HK-2 can prevent ANF and SGN at much longer survival times. Future studies aimed at quantifying the degree of ANF and SGN degeneration might benefit from the use of more sophisticated stereological methods for detecting subtle differences in the degree of ANF and SGN degeneration with the various treatments used in this study^57^.

### Conclusion

Our blast trauma data extend and elaborate upon on our earlier preclinical study showing that orally-administered HK-2 not only protects against conventional NIHL induced by moderate intensity workday noise exposures^18^, but also extremely intense blast wave exposures. HK-2, conveniently administered in rat chow, significantly reduced blast-induced OHC and IHC loss by ~ 67% and ~ 57% respectively. Our results show for the first time that HK-2 completely prevented the blast-wave induced degeneration of ANF and SGN out to at least 60 days post-exposure. Our blast exposure selectively destroyed approximately one-third of the vestibular hair cells located in the striola region of the sacculus, but failed to cause hair cell degeneration in the marginal region of the sacculus, the striola and marginal regions of the utricle and hair cells in the ampulla of the three semicircular canals. The stability and ease of administration of HK-2 make it a promising candidate for human clinical trials among individuals routinely exposed to high levels of occupational and/or recreational noise such as military personnel, musicians and first responders. Because HK-2 passes through the blood–brain barrier, it has the potential to protect against many other sensory and neural injuries that arise from excessive or prolonged oxidative stress (e.g., concussion, drug toxicity, Alzheimer’s, Parkinson’s and aging)^58–60^.

## Methods and materials

### Subjects

The twenty-four male Sprague–Dawley rats (Charles River Laboratories) used in this study were ~ 12 weeks old at the start of the experiment. The rats were housed in the Laboratory Animal Facility at the University at Buffalo and given free access to food and water. The colony room was maintained at 22 °C with a 12-h light–dark cycle. Rats were physically examined at the start of the study for potential health issues. Only healthy looking rats were included in the study; any that appeared unhealthy were excluded from the study.

### Declarations

All procedures regarding the use and handling of animals in research were reviewed and approved by the Institutional Animal Care and Use Committee (IACUC) at the University at Buffalo and all the experiments were carried out in accordance with the Guide for the Care and Use of Laboratory Animals in research. All the performed procedures and all the reported data were in accordance with the ARRIVE guidelines.

### Blast wave exposure

All rats were exposed to 6 blasts presented once every 5-min using equipment and procedures similar to those previously described^52,61,62^. Rats in the four treatment groups (B, BEP, BHK-2 and BEPHK-2) were blast-exposed one at a time. The order of treatment was randomly varied among the four groups, followed by the next group of four in random order. Each rat was anesthetized with an intraperitoneal injection of ketamine (50 mg/kg) and xylazine (6 mg/kg) and stable anesthesia was maintained with supplementary half-doses of anesthetics as needed to maintain a stable anesthetic state. Each anesthetized rat was placed in wire-mesh cage 5 cm with the snout of the rat facing the front of the blast tube opening. The average blast peak pressure level was 186 dB peak SPL (± 0.8 dB, SEM, ~ 39.9 kPa). The intensity was selected based on our prior study in which we found no evidence of tympanic membrane or ossicular damage in rats exposed at 188 dB pSPL^52^, consistent with an earlier report^63^.

### Experimental groups

The rats were randomly assigned by lottery number to one of four experimental groups (n = 6/group): (1) Blast alone (B), (2) Blast plus earplugs (BEP), (3) Blast plus HK-2 (BHK-2) and (4) Blast plus earplugs plus HK-2 (BEPHK-2). All rats had free access to water and standard lab chow except for the BHK-2 and BEPHK-2 groups that also received HK-2 as noted below. The untreated rats in the B group served as the control group against which to evaluate the efficacy of the treatments employed in the other three groups: BEP, BHK-2 and BEPHK-2. The size of the experimental groups was based on a previous experiment in which a sample size of six was sufficient to identify a significant protective effect of HK-2^18^ and/or earplugs^64^.

### Earplugs

Both ear canals of rats in the BEP and BEPHK-2 groups were plugged with a thin wedge of foam cut from a commercial EAR^*R*^ earplug and then covered with petroleum jelly. This ear plugging methods was highly effective at preventing hair cells loss as indicated by data in the results, consistent with our earlier study in which custom earplugs similar to those employed here elevated auditory brainstem response thresholds (a biological estimate of sound attenuation) from 23 dB at 6 kHz to 50 dB at 32 kHz^65^.

### HK-2 synthesis and administration

HK-2 was synthesized and evaluated for purity (> 99%) as described previously^66^. Rats in the BHK-2 group and BEPHK-2 group were fed the same standard laboratory rat chow as the other two groups, however, the chow had been treated with HK-2 as described previously^18,67^. HK-2 treated food was administered to rats starting 7-days prior to the blast wave exposure and continuing for 30-day following exposure. Based on the rat’s daily food consumption, measured every 2 days together with body weight, the average oral consumption of HK-2 was ~ 50 mg/kg/d^18,67^. In our previous study, we found that 40 mg/kg/d was highly effective at preventing hair cells loss from prolonged exposure to 8 h/day noise. Because the blast exposure used in this study was more intense, we attempted to enhance its efficacy, by utilizing a dose that was 25% higher than the one used previously^18^. Previous studies reported a delayed production of noise-induced oxidative stress peaking ~ 10 day post-exposure and still present 14 days post-exposure^16^. Therefore, we administered HK-2 for 30 days post-blast to enhance its efficacy; this post-exposure treatment duration was three time longer than in our previous study^18^.

### Tissue preparation

Approximately 60-days after the blast wave exposure, the rats from all four groups were deeply anesthetized with ketamine (50 mg/kg, i.p.) and xylazine (6 mg/kg, i.p.) and decapitated. The temporal bones were quickly removed. Under a dissection microscope, openings were made in the cochlear apex, round window, and the oval window. Samples for surface preparations of the cochlear basilar membrane and vestibular end-organs were perfused with 10% formalin in phosphate buffered saline (PBS) into the cochlear and vestibular cavities and then immersed in the fixative overnight as described previously^67,68^. Samples for temporal bone sections were fixed with 2.5% glutaraldehyde in 0.1 M PBS for 6 h. After rinsing with PBS, samples were immersed in 2% osmium tetroxide in 0.1 M PBS for 2 h^69^. To avoid experimental bias, the researcher conducting the anatomical analyses was blind to the experimental treatment. Only the researcher administering the blast exposure was aware of the treatment a specific rat received.

After fixation, samples for surface preparations of the cochlea and vestibular end-organs were decalcified (Decalcifying solution-Lite, Millipore Sigma, D0818) for 48 h. Then, the cochlear basilar membrane, macula of saccule, macula of utricle, and three crista ampullae were dissected out under a dissecting microscope, and stained with Harris hematoxylin solution, and mounted in glycerin on glass slides as described previously^68^.

Samples used for temporal bone sections were decalcified with 10% EDTA solution for 5 days, dehydrated through a graded series of ethanol solution ending at 100% and pure acetone as described previously^69^. The temporal bones were then embedded in Epon 812 resin. After high temperature polymerization, the temporal bones were cut parallel to the axis of the cochlear modiolus at a thickness of 3 µm using a ultramicrotome (Reichert Supernova) equipped with glass knives. The semi-thin slices were collected on glass slides, and stained with toluidine blue.

### Cochlear hair cells

The entire cochlea was removed and mounted in half-turns as a flat surface preparation in glycerin on glass slides and coverslipped. The hematoxylin stained cochlear basilar membrane was examined over its entire length with a light microscope (Zeiss Standard, 400× magnification). Cochlear IHC and OHC were counted along successive 0.12–0.24 mm intervals from the apex to the base of the cochlea. A hair cell was counted as present if both the cuticular plate and nucleus were clearly visible and considered missing if either were absent. The percentages of missing IHC and OHC were determined based on laboratory norms for normal adult rats^70^. The cell counts were entered into a custom computer program to generate a cochleogram showing the percentages of missing OHC and IHC as a function of percent distance from the apex of the cochlea based on laboratory norms as described previously^67,68^. Cochlear location was related to frequency using a rat frequency-place map^26^. Mean cochleograms (n = 6/group) were computed for each group using Excel and GraphPad Prism software^68,71^. Mean percentages (+ /− SEM) of OHC and IHC losses were computed over 20% intervals and plotted as a function of percent distance from the apex and related to frequency.

### Auditory nerve fibers (ANF) in habenula perforate

Sections were taken from the Epon 812 embedded samples tangent to the habenula perforata as described previously^72,73^. Sections were mounted on glass slides, stained with toluidine blue and examined under a light microscope (Zeiss Axioskop) at 200X magnification. Specimens were photographed with a digital camera (SPOT Insight, Diagnostic Instruments Inc.) and processed with imaging software (SPOT Software, version 4.6; Adobe Photoshop 5.5). For each experimental condition, the number of ANF was counted in each habenula perforata in the middle of the basal turn (~ 70%-distance-from-apex, 25 kHz region). Counts were obtained from 10 habenula perforata in the middle of the basal turn and a mean value computed at that location for each animal. ANF counts were obtained from 6 cochleae per group.

### Spiral ganglion neurons (SGN) in Rosenthal’s canal

To quantify the number of SGN in sections, 3 µm thick serial sections were cut parallel to the axial axis of modiolus. Sections were mounted on glass slides, stained with toluidine blue, examined under a light microscope, photographed and processed using imaging software as described previously^72^. For each cochlea, representative photomicrographs of Rosenthal’s canal were obtained from the middle of the basal turn (~ 25 kHz location). The number of SGN were counted in each section (every fifth section) of Rosenthal’s canal. SGN counts were obtained from five separate sections from each animal and a mean value was computed. SGN counts were obtained from 6 cochleae per group.

### Quantifications of vestibular hair cells

The hematoxylin stained vestibular sensory epithelium of the macula of the saccule, macula of the utricle, and three crista ampullae were mounted in glycerin on glass slides as flat surface preparations. The samples were coverslipped and examined with a light microscope as described previously^71^. Section were examined under a light microscope (1000×) and the number of vestibular hair cells were counted in a 0.0108 mm^2^ region (see Fig. 5A). Hair cell counts were obtained from four regions in each vestibular sensory epithelium and a mean value was computed for each vestibular end-organ. Vestibular hair cell counts were based on the presence of a darkly stained nucleus. Many, but not all, of the darkly stained vestibular nuclei were surrounded by lightly stained calyces.

### Analysis

GraphPad Prism (ver. 5) software was used to plot all the numerical data in the graphs and to statistically analyze the results.

## Data Availability

The datasets used in this study are available from the corresponding author on reasonable request.
